# The Changing Process of Disability Identity: A Trajectory Equifinality Model Analysis of Japanese with Physical Disabilities

**DOI:** 10.1007/s12124-025-09953-0

**Published:** 2026-03-03

**Authors:** Masakuni Tagaki

**Affiliations:** Graduate School of Sustainable System Sciences, Osaka Metropolitan University, Osaka, 599-8531 Japan

**Keywords:** Disability identity, Disability community, Narrative, Trajectory Equifinality Model, Japan

## Abstract

This study examines the development of disability identity in Japan’s disability community. Semi-structured interviews were conducted with three Japanese participants with physical disabilities who had active interaction with others with disabilities. The interview transcription was analyzed using the KJ method and the Trajectory Equifinality Model developed in Japan. The analysis showed that the participants coped with a sense of isolation from society and experienced dilemmas about their invisible disabilities. They broadened their views from personal to general disability issues at several turning points. The process was not linear; they examined the unprocessed path deeply to understand the meanings of actual experiences after each turning point. They were influenced by family members, people with similar or different disabilities, people without disabilities, and local government officials. In addition, their cultural and structural environments either encouraged or hindered their problem-solving abilities and development of new viewpoints. Their relationships with other people with disabilities enabled the participants to broaden their perspectives on disability issues and realize the limitations of their own activities. They were not satisfied with their achievements and neglected to cultivate successors. The study results demonstrated that forging and sustaining a disability identity does not mean people with disabilities belong entirely to the disability community. Instead, they move between the disability and non-disability communities. That holistic identity gives people with disabilities the opportunity to seek new activities using a broader imagination.

## Introduction

### Approaches to Physical Disability Experiences

Studies on physical disability experiences can be divided into two types (Tagaki, 2016a).^1^ The first encourages people with disabilities (PWD) to accept their status, adjust to psychological issues and bodily malfunction, and reframe their entire lives to assume a desirable psychological status. The second is for those who sustain disabilities over the long term, in which research seeks the meaning of disabilities and does not assume a desirable psychological status. The rigorous definition of “long-term” is not available. However, “long-term” in this context means that the disability, while chronic, is stable after the application of acute medical treatment required by the onset of the disability; however, regular medical checkups and medication are required and the health of people with a certain type of disability may deteriorate. Tagaki (2016a) found that people sustaining disabilities in the long term revise the meaning of their disabilities as they lead their lives through changing contexts. PWD have difficulties with bodily malfunctions and social activities, which cause challenges in employment and critical attitudes of people without disabilities toward them. However, PWD value relationships with others with similar disabilities because they can obtain advice for their impairment, assistance devices, and disability services. They also expand their views on disability issues that differ from theirs by meeting those with diverse disabilities, who regard their experiences as unique because of their disability. However, these experiences are rarely examined.

The current study aimed to examine the process and focal points of people sustaining physical disabilities in the long term as they establish relationships with others with different disabilities, both by participating in disability-related activities and by seeking ways to transmit their achievements through the disability movement. The theoretical framework is disability identity with generativity (Erikson, 1959), which refers to PWD viewing disability as a positive and essential aspect of their lives and having a sense of solidarity with PWD in the disability community (Forber-Pratt et al., 2020; Tagaki, 2025). Because the generativity theory has a wide meaning, the current study defines it as the process by which older PWD convince younger generations to continue their achievements.

Disability identity is a noteworthy concept describing how PWD develop their identities based on the experiences of others living with a variety of disabilities. Disability identity is theoretically grounded in social identity (Hogg & Abrams, 1998), in which people regard themselves as members of a group consisting of individuals with similar attributes by considering the favorable characteristics they share.

Tagaki (2025) remarked on two components of disability identity for PWD: decision-making at important events and a counterculture against socially dominant norms such as productivity and efficiency in the labor market and academic achievements being a prerequisite for climbing the career ladder. To foster these components, PWD share among themselves the discriminative experiences in their social lives to focus on a target to combat. The mutual exchange of these experiences also engenders a strongly favorable attitude toward advocacy and political movements (Reher & Evans, 2025). Examples of these experiences include limited access to education, transportation, and unfair employment practices. Advocacy activities can be conducted offline and online; thus, belonging and contributing to the disability community does not always have to involve in-person activities.

Over time, different generations of PWD have engaged in varying advocacy and political movements. Generativity is helpful when discussing the contribution of older generations of PWD to younger members in their community. It also calls for respecting differences among generations in terms of social attitudes and disability policies (Andrews, 2020). Notably, Erikson (1959) did not elaborate on disability identity, and previous studies have not examined the generational issues involving PWD’s contribution to the disability community. However, contributing to the disability community is a big concern for older generations. The current generation realizes the importance of disability issues in social models but does not recognize how they are involved in disability-related activities. One reason is that the current generation has lived in an improved society owing to the Americans with Disabilities Act and thus has different interests, including non-disability matters (Forber-Pratt, 2019). Wu et al. (2024) remarked that PWD may wonder whether their personal experiences contribute to disability policies. Instead, they should appreciate their connections to non-disability-related activities. The current study does not control for the timing of the onset of the disability because it does not aim to compare which onset group (e.g., congenital vs. acquired disabilities) achieves a certain psychological status such as adapting to the disability and greater life satisfaction (Tagaki, 2016a). Instead, the goal is to describe the meanings of disabilities through the narratives of PWD. Furthermore, previous studies lack consensus on which group shows better psychological adjustment. People with congenital disabilities have often adapted to their disabilities and view themselves positively (Bogart et al., 2012; Li & Moore, 1998). However, they face stigma and social isolation in schools and struggle with subjects such as physical education, which can force them to regard their disability more negatively (Bogart et al., 2019; Magill-Evans & Darra, 2011).

On the contrary, people with acquired disabilities have lost their bodily functions and cannot manage social activities or networking (e.g., Bogart, 2014); they also show more depressive tendencies than people who have congenital disabilities (Kim & Park, 2024). However, they can cope with difficulties resulting from their disabilities by using their experiences and reorganizing resources before the onset of the disability (Bogart, 2020) and emphasize new experiences thereafter (Tagaki, 2016a). Based on these conflicting findings, we cannot confidently determine which timing of the onset of the disability correlates with a better psychological process, further justifying our focus on the descriptive experience rather than on a comparative analysis.

### Trajectory Equifinality Model

This study employed the Trajectory Equifinality Model (TEM) (Sato et al., 2006) to show the multilinear pathways to a specific goal. The TEM is a commonly used method for various topics, including disability research, to depict the diverse processes involved in pursuing a specific goal. Using the TEM, Katsuya and Sano (2021) showed the multiple paths through which people with hearing impairments disclose their conditions.

The TEM does not show a mere life course; instead, it shows the diversity of routes to a goal (Lyra et al., 2018). Broadly, the model consists of a goal, focal points along the diverse routes, and the mutual relationship between the actor and the outside world. First, the TEM employs irreversible time (Sato et al., 2006), which can have several meanings, but the main one is sequential time that does not move backward and cannot be measured by a clock.

Second, the model is based on the Equifinality Point (EFP) (Sato et al., 2006), which is a convergent or equal arrival point. Although each person selects different directions at important junctions, they arrive at the EFP, which can also be thought of as a goal. The EFP is also a focal point that researchers can determine operationally (Kojiro et al., 2019). However, this focal point is neither fixed nor idealized; it depends on the social and cultural context of the designated researcher.

Sato et al. (2006) created the Polarized EFP (P-EFP), which shows the contradictory and complementary pathways to reaching the EFP. These researchers intended to prevent the EFP from becoming the dominant and valued goal. Depicting events and alternative paths can clarify researchers’ self-evident conclusions and widen the existing EFP. Moreover, the P-EFP forces researchers to revisit the presumed goal and the progressive narrative that one’s bad experiences become better ones by overcoming adversity (Tagaki, 2016b).

Third, the TEM suggests several focal points that depict an individual’s rich experiences and diverse pathways before arriving at the EFP. According to the TEM, individuals proceed along multiple paths through several important Bifurcation Points (BFPs), negotiating with the external environment. The Obligatory Passage Point (OPP) is an event that most individuals inevitably experience in a specific cultural, societal, and biological context if they attempt to reach the EFP. The OPP can also be a BFP. The BFP is the opportunity for an individual to choose one trajectory or its opposite. An unselected trajectory can be essential for deepening the understanding of the selected trajectory. The second EFP occurs when the person arrives after selecting the previous EFP.

The TEM is a tree of real and imagined options in life (Menhart, 2024). The model establishes specific concepts to depict varied routes toward a specific goal. The TEM values the presumption and uncertainty in the multiple paths to the EFP by actively assuming unknown paths. This approach differs from studies on turning points (e.g., Mandelbaum, 1973), which do not observe unchosen trajectories. The TEM holds that meaning-making processes can deepen the dynamics between real and imagined worlds (Lehmann et al., 2020). The model is not used to manage only the past and present. It differentiates between the past and the future by determining actual and potential paths, as an actor might have elaborate experiences at focal points that were considered but not pursued (Menhart, 2024).

Regarding an actor’s experiences, Societal Dominance (SD) and Societal Guidance (SG) are significant concepts for examining actors’ negotiations with the outside world. SD hinders reaching the EFP, but SG supports and encourages individuals to arrive at it. The author can assume many things such as SG from the micro to macro levels provided that human development progresses at multiple levels. For example, at the micro level, the author can consider families, professionals, other PWD, and disability services. At the macro level, SG includes disability policies such as the UN Convention on the Rights of Persons with Disabilities, the Japanese Disability Discrimination Act (Act No. 65 of 2013), and general media discourse on disabilities.

The same phenomenon can be SD or SG depending on the context and timing. For example, family members are expected to be caregivers for PWD in Japanese society, but they could hinder PWD from leaving their homes to live by themselves, and caregivers could be fatigued by the overwhelming burden. SD and SG influence a stable process in which no changes are found, but the person actively negotiates outward, recognizing SD and SG. For example, PWD interact with disability policies, general attitudes, and the public discourse.

The model employs Valsiner’s (2007) view of narratives that recount the past through interactions with society, culture, and interference; narratives can generate the meaning of experiences in the future. People cannot help but consider social and cultural norms when choosing a certain direction. The shadow or trajectory not taken can reveal the self-obvious value in the selected path. From the perspective of time, the TEM reveals how people’s lives diverge at focal points and converge to arrive at the EFP (Zittoun & Gillespie, 2016). Uncertainty cannot be avoided if an unchosen path is actively examined; however, uncertainty can offer flexibility during unexpected catastrophic events in their lives. Tateo (2018) noted that the narrative approach to “health” matters risks adhering to a peculiar normative narrative and dismissing alternatives.

Disability identity is part of the meaning of disability narratives (Andrews, 2020). As mentioned, the narrative is not merely a recall and interpretation of past events but a referral to the future. Zittoun (2020) remarked that imagination is required to refer to the future through communication with others and social representations, including social movements. The author argues that Zittoun’s (2020) perspective can include disability policy, disability discourse in the media, and social networking services that contribute to the disability identity narrative.

## Method

### Participants and Recruitment

Three participants (Participant A, Participant B, and Participant C) were selected from the 11 participants of a previous study (Tagaki, 2025). Those 11 participants were recruited from local disability support groups in western Japan using purposive sampling (Teddlie & Yu, 2007). The inclusion criteria were as follows: (a) had engaged in disability-related activities for several years, (b) had any type of physical disability, and (c) were able to attend the interview in person. The author contacted individuals via phone, e-mail, and in person to explain the purpose of the study and obtain written or verbal consent to participate based on their preference. The participants were aged 40–70 years (*M* = 58) and the time since “the onset of the disability ranged from 15 to 60 years (*M* = 23). The ethics committee of the university with which the author is affiliated approved the study protocol.

The three participants with mobility or visual impairments were in their 50 s and 60 s at the time of the interviews. The selection criteria for the participants were as follows: (a) had engaged in disability-related activities for several years, (b) could describe their disability experience after onset, and (c) could described the precursors and successors of their disability-related activities. The last two criteria were emphasized for the purposes of the current study. Age at the onset of the disability was not controlled for to elicit possible similarities and differences.

The participants’ background information is presented in Table 1. Each participant had been issued an official disability identification card from the Japanese government based on the Act on Welfare of Physically Disabled Persons (Act No. 283 of 1949); the card indicated the most severe degree of disability according to medical doctors’ reviews on the impairment. The disability support needs grade comes from the Act on Providing Comprehensive Support for Daily Life and Life in the Society of Persons with Disabilities (Act No. 123, November 7, 2005). Disability support differs from impairment grade in that it shows an individual’s degree of need for social services in daily life. Participant B was not assigned a disability support evaluation because care services for people with visual impairment were available without disability support.

**Table 1:** Participants’ profiles

	Ages	Disability-onset ages	Disability type	Employment status	Living status	Daily support need grade
A	60	20	Mobility	Welfare-related	Single	6
B	50	0	Visual	Welfare-related	Family	-
C	50	40	Mobility	Employment	Family	5

### Data Collection

Semi-structured interviews were conducted twice with the participants, with their permission. The average duration of the two interviews was 230 min. On average, each interview was transcribed into approximately 65,000 Japanese characters. The interviews were conducted between August 2016 and May 2017 at local disability centers that were convenient for the participants. The interview method was based on the life story interview technique (Atkinson, 2001) because the TEM could be mentioned as part of a life story study, which is a suitable method for elaborating on participants’ long-term experiences (Tagaki, 2016a) with follow-up questions.

At the beginning of the interview, the author obtained basic details on the participants’ situations, including the present state of their disability, utilization of medical and welfare services, means of transportation, and trajectories of their core daytime activities such as medical rehabilitation, vocational training, and employment.

The participants talked about life events such as when they became aware of their disability, admission or graduation from school, employment and marriage, the relationship between hospitalization and disability, and changes in their lives due to aging.

Questions about disability pursuits included inquiries about how they became interested in other PWD, became involved in their current activities, developed mutual understanding and solidarity in terms of the various disabilities among members, used their disability-related experiences in their daily lives, and started training younger members.

### Data Analysis

The author summarized the transcriptions using the KJ method (Kawakita, 1967) before depicting the TEM diagram based on an analysis guide (Arakawa et al., 2012). “KJ” came from the founder of this method “Kawakita, Jiro”, a Japanese anthropologist. Kawakita (1967) adapted this method from Peirce’s notion of abduction, relying on intuitive, nonlogical thinking processes. The KJ and grounded theory methodologies (Glaser & Strauss, 1967) are similar in that they both develop connections between categories consisting of codes generated from segmented text data. However, the goal of the KJ method is to create new connections between contradictory or illogical ideas or concepts. By contrast, grounded theory aims to develop a logical integration of categories into a theory.

First, the transcripts and memos were carefully reviewed to understand each participant’s life story. Second, the sentences in each transcript were compiled into basic semantic units (generally three or four sentences), resulting in approximately 450 units for each participant. Third, the author provisionally grouped similar units for each participant with abstract labels that characterized the grouped units (e.g., new units). If a unit was not semantically similar to other units, it was left as is.

Fourth, the author grouped and organized both the original and new units. The grouping and labeling procedures were conducted approximately three to four times, and the final unit was divided into those that could be included in the timeline and those that could not, unlike the usual procedure of the KJ method. On average, 15 categories were obtained for each participant. The author used Atlas.ti Windows (version 9.0) developed by Scientific Software Development GmbH (Germany).

**Fig. 1:**
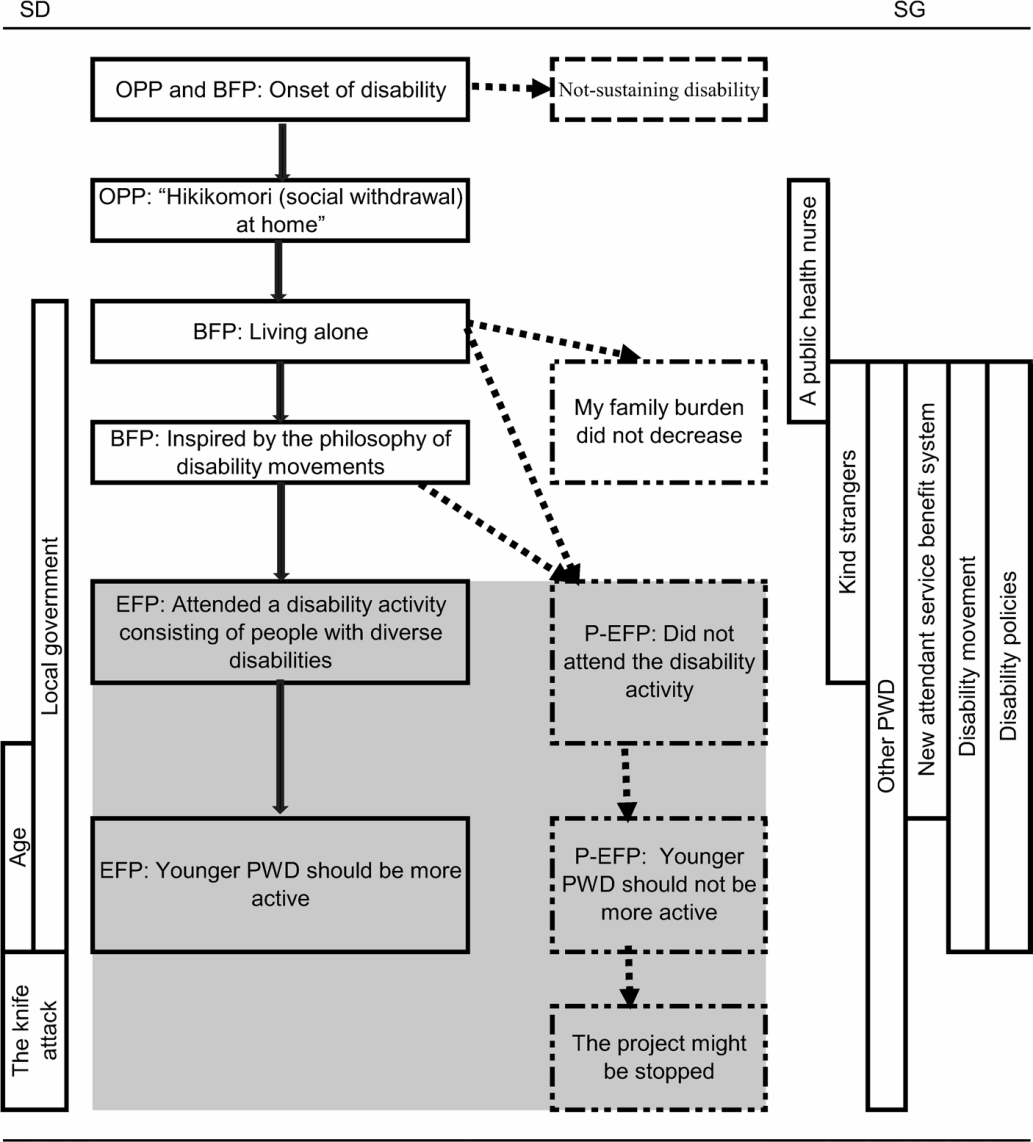
Participant A’s TEM diagram

**Fig. 2:**
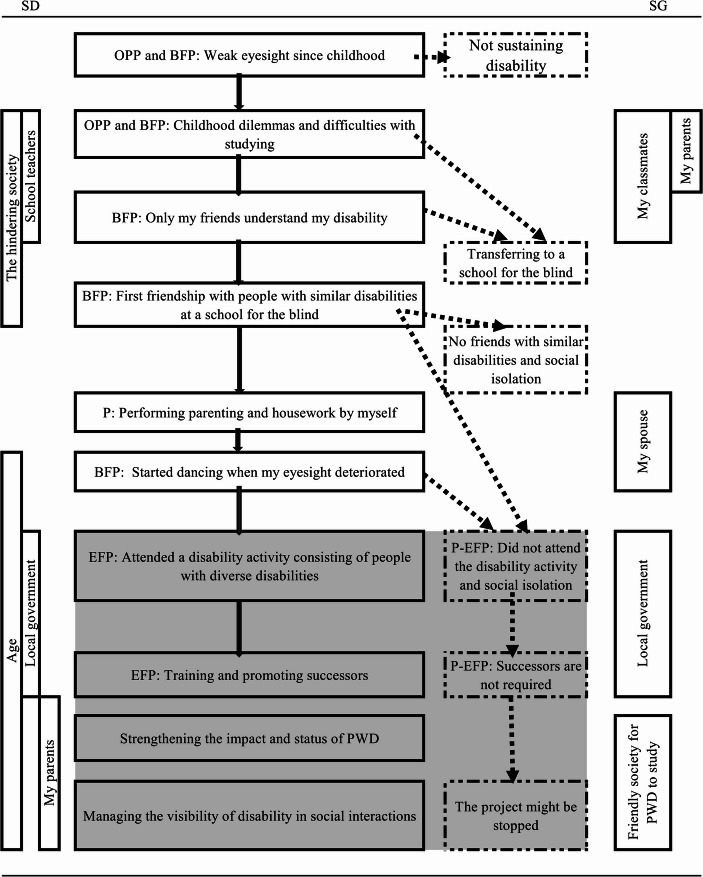
Participant B’s TEM diagram

**Fig. 3:**
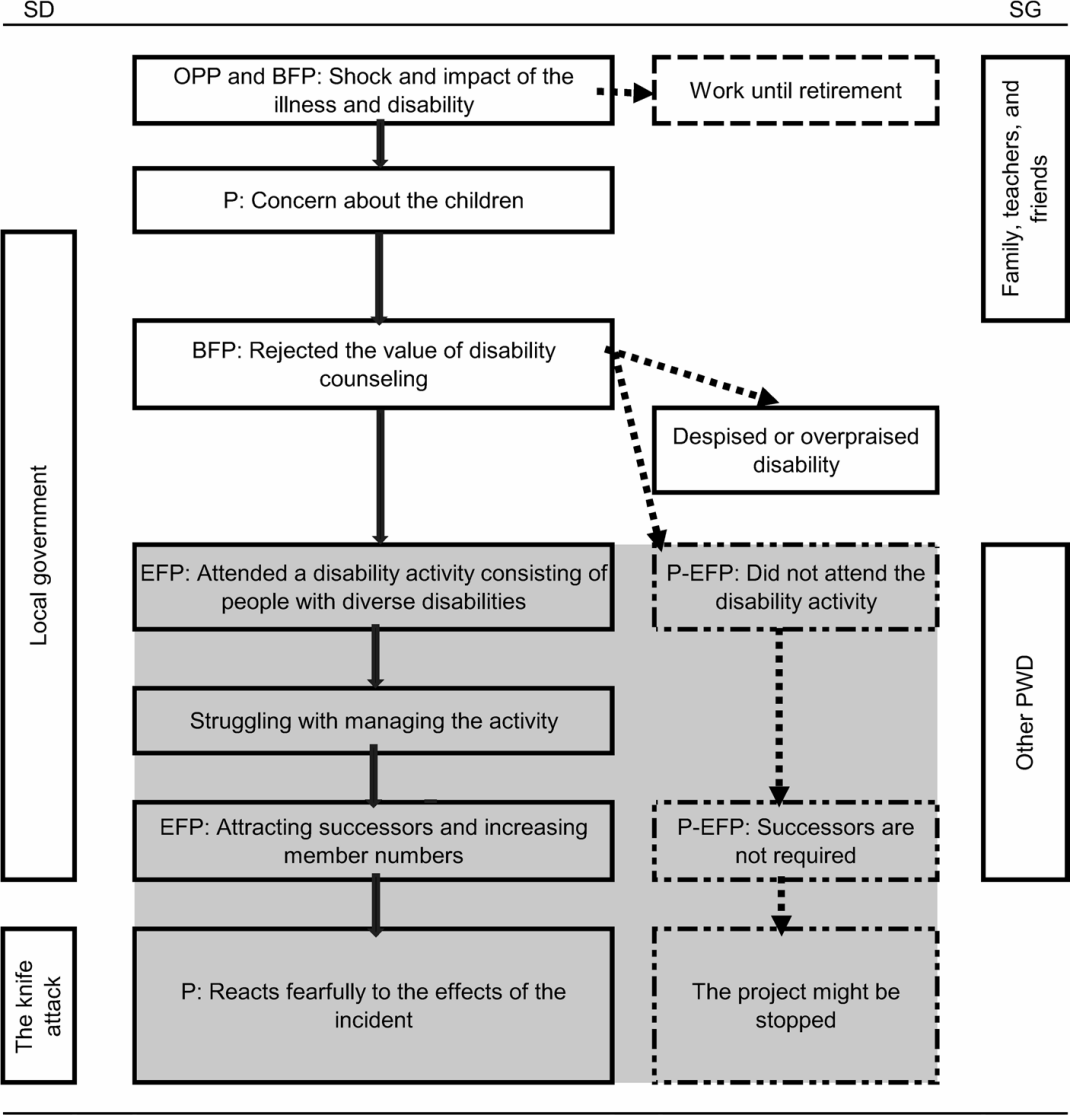
Participant C’s TEM diagram

As the TEM diagram (Figs. 1, 2 and 3) aims to visualize the process of the ongoing equilibrium status (Sato et al., 2006), all the relevant grouped units were arranged on a timeline and the relationships among the units were organized. The diagram depicts the participants’ changing attitudes toward their disabilities, relationships with other people with similar and different disabilities, disability-related activities, and continued struggle with diverse issues. The solid lines in the figures show the paths the participants took. Based on TEM theory, the dotted lines indicate possible paths that remained untaken, as can reasonably be assumed from their experiences, some of which were mentioned by them. The figures also highlight the background and context of local government officials’ attitudes, disability policies, and related discourse. The area of the EFP, the P-EFP, and related experiences is highlighted in gray to show them as a zone. The SD and SG are vertically depicted to clarify their coverage.

## Results

### Results for Participant A

#### OPP and BFP: Onset of Disability

The patient sustained a spinal cord injury during a road traffic accident. He realized that his injury could not be cured, as he saw other patients with a similar status to his during hospitalization. His family, who was informed by his doctor, informed Participant A of his status. He did not elaborate on what his life might have been like had he not sustained the injury.

#### OPP: Hikikomori (social withdrawal) at Home

After leaving hospital, he said, “I didn’t really leave home (from the 1970 s to the late 1980 s).” Although no social services were available, his brother was prepared to take care of him. During hospitalization, he met other patients who had sustained spinal cord injuries; they visited him at home, but he could not go out with them. He avoided meeting friends he had known before the disability. He said, “If I go out with them now, I will drag them down.”

#### BFP: Living Alone

A public health nurse and an older person with a disability recommended preparing Participant A to live alone in the late 1990s. He wanted to “free” his family from the burden of caring for him and did not want to live in an institution.

Besides the difficulties of transferring from bed to wheelchair, the biggest problem was securing the 550 hours of caregiving needed to live alone from the official in the town where he lived. He said that the local government was opposed to people with severe disabilities living alone because of the additional budgetary burden. “I felt (the local government) was very concerned that if many of these people came out, it would be a (financial) problem.”

He was the first person with a disability who lived alone in his city with a caregiver. He added “Absolutely. There were no PWD in S-city who had such a concept of living alone.”

His house looked the same as when he was not injured, but somehow, it was different. “The smells and sounds around the house were amazing… I worked under the car (pre-injury). I never thought that the sound of the engine was too loud, but the engine of the parked car was too loud… It is like being Urashima Taro.”

In those days, social attitudes toward people in wheelchairs were worse than today. It was uncommon to see a person in a wheelchair around town and would result in a call to City Hall or the Health Department. Participant A stated that PWD and their families generally believed that they could not live alone. “(Talking about living alone) is eye poison, ear poison (socially unacceptable).” However, “(In this town), some onlookers assisted me when they saw me arguing with the station staff. They said, ‘Brother, what are you doing? I see, you want to get on the train’ (and) they helped me.”

#### BFP: Inspired by the Philosophy of Disability Movements

Participant A was motivated to join the disability movement with other PWD by attending workshops conducted by disability organizations. He learned the importance of working collectively. For example, he understood that “demonstrating in front of a bus is required to seek social attention and improve accessibility to public transportation,” even though demonstrating might be regarded as too excessive. In addition, he was worried about his parents aging as he grew older. He said, “Parents need to think about what will happen to their children after the parents’ death, but (they do not).”

#### EFP: Attended a Disability Activity Consisting of People with Diverse Disabilities

He joined a disability activity to introduce his organization to other PWD and broaden his views on disability issues. During this activity, he built relationships with many different types of PWD, such as those with visual impairments and mental disorders. “It was good that we got to know people from different fields during the activity.” He added that concrete activities by PWD were more important than discussing disability issues alone.

#### EFP: Younger PWD Should be More Active

Participant A’s concern was that he and his friend from his disability activities had not identified or trained successors because the same people continued to lead these activities. Younger members were reluctant to assume active roles because they participated in activities only when requested by other PWD. He said, “We did not do a good job of replacing them with (younger members); we didn’t invite younger PWD to join.” He added that young PWD complain about disability issues but do not take an active role. Nevertheless, he envisaged disability activities in which younger PWD actively participated. “The more I see how active a certain city is, the more I wonder why we cannot do it that way.” He expected several leaders to harmoniously manage the activities of the younger PWD, rather than rely on one person. However, although he and his colleagues started many activities for younger PWD, the majority ended up as one-offs.

Although Participant A had succeeded his predecessor to run the disability support center, he could not find his own successor. PWD who were active in the past had died. Moreover, his older friend with disabilities, who had established the center and had helped Participant A live alone, was now busy advising other PWD on living alone, as he had done many years ago. In addition, current young PWD seemed to think that the disability movement was “unfashionable.”

He stated that people with different disabilities visited the center, such as people with visual and hearing impairments, although some staff members were interested in people with acquired mobility impairments. He added that people with spinal cord injuries were classified as having different disabilities from those with the same type of disability. Some were aware that the incidence of spinal cord injuries was higher than that of cervical cord injuries. He said that a successor could change the center’s name if he or she wished. He said, “After I quit (as a representative of the center), I will not care if it is not called (current name focusing on living alone); it can be called the Disability Counseling Center.”

The Center’s activities were aimed at collaborating with the local government rather than negotiating with them. However, when using various services, they liked to highlight problems. He said, “If you use multiple services, you will find many things that are not right.” However, he was frustrated by the few opportunities to address these issues available to them. He said, “The city was not interested in such negotiations.”

Participant A regretted that PWD had become accustomed to discrimination. For example, if a station attendant refused to help a person with a disability get on a train, the person said nothing. His remark indicated that the Disability Discrimination Law did not enable PWD to express their discrimination experiences. He expected them to insist on publicizing the unfair experiences they had. Moreover, he expected new members to become interested in knife attacks against PWD at institutions. He remarked that they must focus on the eugenics philosophy that was implicitly accepted without considering that a man with a mental disorder was convicted of a knife attack in Kanagawa prefecture in 2016.

At the age of 65 years, he also regretted what he had been unable to do. Compared with young people today, he did not have the power to act. He said, “When I think about it, it is very dark.” When he had made a plan to use welfare services for the disabled and had brought it to the local government office, able-bodied people had been “credited” more than those with disabilities.

At this point, however, his active disability pursuits ended because his physical strength was declining. He began experiencing physical limitations. He could no longer remember people’s names and needed more time to recover from pressure sores. He said, “I wonder if I am going to ‘expire’ by this spring or so. Therefore, I will have to spend less time in a wheelchair (because I don’t have the strength anymore). In retrospect, I realized that because I could not use a wheelchair, I am a failure, a disqualified person.”

### Results for Participant B

#### OPP and BFP: Weak Eyesight Since Childhood

The onset of Participant B’s weak eyesight was unclear. She was “flagged” at a vision test in elementary school. Although her impairments currently have a diagnosis, then they were referred to as congenital amblyopia. She said, “We didn’t know (about my eyes and disease) then, and we don’t know now. We do not understand our own eyes. The doctors did not explain it to me, and they did not understand it either.” She did not describe in detail what her life without her impairment would have looked like.

#### OPP and BFP: Childhood Dilemmas and Difficulties with Studying

“When I was halfway through my childhood, I had a dilemma, lots of dilemmas.” She did not carry a white cane, and it was difficult to observe her disability from the outside: “It was bothersome to explain my disability.” She recounted that the dilemma was severe during her school days because she was considered strongly nearsighted. Nevertheless, she went on school trips and excursions as usual. Studying was difficult at her regular junior high school, even though the current learning environment was better, such as text-to-speech applications and diverse support at admission examinations. The homeroom teachers did not understand her condition and thought that she could see if the letters on the blackboard were enlarged. She did not receive any extension in the time allowed for regular examinations. She said, “In those days, if you said something like that, you were told to go to a school for the blind. It was tough for my eyes to comprehend what I was seeing, but I was able to walk normally” However, the writing on the blackboard gradually became increasingly difficult to see.

She underwent a reading examination without braille. She could not read all the sentences, saying, “I could not complete the test because I couldn’t read… I am sure I was (frustrated and pathetic) anyway.” She did not achieve pass marks on the exam.

#### BFP: Only my Friends Understand my Disability

Her classmates recognized her troubles at school. For example, in home economics, they helped her thread needles. She said, “If I had been bullied in class or something, I might have considered going to a school for the blind (before high school).”

#### BFP: First Friendship with People with Similar Disabilities at a School for the Blind

After regular high school, she attended a school for the blind where she shared her difficult experiences with visually impaired classmates. Studying became more manageable after she realized, “This enlarged textbook is hard to read. I have to learn braille.” She said, “I realized there how bad my eyesight was.”

#### P: Performing Parenting and Housework by Myself

Participant B continued, “I do my housework and childcare with a few assistants… although it takes me a long time.” She raised her child without outside support. For example, she took her child for immunizations and physical examinations. Her child became aware of her mother’s disability in kindergarten. She said, “In hindsight, I should have been less strict and asked her to help me.” Her husband helped her check the small print in documents from school, similar to when he helped with bottle-feeding.

#### BFP: Started Dancing when my Eyesight Deteriorated

When her eyesight weakened, and she could no longer move around on her own, she participated in dance workshops that allowed her to express herself. She said, “It changed the way I look at things. Being involved in dancing made me realize how important it is for PWD to be active. I developed a strong mindset and the ability to go anywhere. Also, dancing brought me into contact with various PWD.”

Her vision deteriorated completely when she was 36 years old. She said, “It was a little tough,” because of the sudden loss of her eyesight. Age was also associated with a decline in physical strength. She said, “There are many things I can’t do anymore… I did not think my eyesight was this bad. I did not know my eyesight would eventually deteriorate this much.” She added, “Motor function also declines, as does intuition, and mine is dull.”

She recounted that her parents had tried to help her live without trouble: “My parents were concerned about exposure and had hidden my visual impairments… My mother would think, ‘I’m so relieved she is married and has children.’” She talked to her parents about her concerns to prevent them from worrying about her.

#### EFP: Attended a Disability Activity Consisting of People with Diverse Disabilities

Before attending her current disability group, she was a member of another group but felt uncomfortable because the group made requests, thinking only about lobbying the government for their own benefit. However, in her current disability group, she has become familiar with people with different disabilities such as mild intellectual and mental disabilities and has tried to communicate with people with hearing impairments. She has learned about people with cervical cord injuries who needed help eating because of hand impairments. She said, “It was interesting to collaborate with people with all kinds of disabilities.” She joined a campaign to decrease the number of illegally parked bicycles and went to schools to talk about disability awareness: “It is good that they are working on their own and contributing to society. It is not just about us or for us.”

#### EFP: Training and Promoting Successors

In her disability group, Participant B wanted to take over the current duties of the new leader. To do so, she needed someone to succeed her. She found an interested newcomer, but that person was “set aside” to work with a “senior” who had been a member for a long time. Participant B wanted to leave the group to avoid taking initiative within it. She engaged in some activities with group members, but not enough. She thought “some people will be active alone, just a few.” She considered three categories: leaders, those who work under their instructions, and those who do nothing.

#### Strengthening the Impact and Status of PWD

Participant B strengthened the group’s PWD initiative. She said, “I have doubts about the local government currently leading the initiative.” Ironically, she used to regard government officials as team members rather than as negotiating partners, saying “the staff member in charge worked (kindly) with me.” Now, however, she insisted that PWD must speak for themselves; she was sad when she observed people with visual impairments taking for granted what others do for them and allowed a female carer to be mistaken for a mother. Participant B conducted disability awareness at schools and talked about her disabilities. At the time, government officials would not get involved in discussions related to PWD as the topic of disability awareness was not considered an issue for people without disabilities.

Moreover, she elevated her status and enjoyed completing college, as she did not have to spare time for her grown child. She wrote about the relationship between people with invisible disabilities and the public in her academic assignment. The university teachers were pleased that people with visual impairments took the course, and found her topic worthy of discussion.

#### Managing the Visibility of Disability in Social Interactions

Participant B says, “Nowadays, we must show (the unique circumstances of PWD).” Meanwhile, people without disabilities “feel that if they write in large letters, they (people with visual impairments) will be able to see them.” In addition, she believes that she should “tell people at the beginning that I have a visual impairment by showing my white cane,” and PWD should be open about their disabilities. Participant B said, “(Even I wonder if I should disclose my impairment), but, at the present time, we (PWD) must inform people about our disabilities for them to know about it… If the times were slightly more advanced, we would interact (with people without disabilities) with no obstacles.”

She avoided being seen as a person with a disability. Her comments about informing people about her disabilities notwithstanding, Participant B pays attention to how she dresses and walks when she goes out with her family: “I don’t like it when people say things like ‘you can’t see, so…’ I think this is because I am a woman. Even if you look, you can’t tell. This is also for the sake of my family. (Being unfashionable) is not good, if I go out with them.” Her walking method changed because of her deteriorating eyesight. However, when talking to people, she still tries to look in their direction, “Even if I can’t see, I like to keep my eyes open, and I try to look in the direction of the person I’m talking to.”

### Results for Participant C

#### OPP and BFP: Shock and Impact of the Illness and Disability

When Participant C found out about her disease in 2000, she was unaware of what had happened. She said, “No test ever indicated that there was anything wrong with me.” She was unable to sleep at night, and experienced pain and anxiety. All she could do was “rest and examine.” She was unable to move her body while in the hospital. The results were not conclusive; however, she had to undergo several surgeries.

Participant C was familiar with the struggles of having disabilities as she had relatives who had them. She could not wear the clothes she wore before the onset of her disability. She was teased for her appearance when she went shopping on a bicycle. She realized that “it is impossible to keep working.” If she had not had this disability, she would have worked until retirement, as initially planned. She said, “I had to give up everything (due to my disease), and then I lost everything.”

#### P: Concern about the Children

She was mentally unstable because she was away from her child and did not know what was going to happen. She wanted to reduce the burden on her child and husband, a devoted caregiver. She believed that if the helper system had been available earlier, her family might have had an easier time.

After discharge from the hospital, she added devices such as handrails to move around her house using a wheelchair. She had to negotiate with the local government to provide disability services such as a carer. The local government did not go into detail about her disability services. She waited a long time to obtain a wheelchair through a public grant. In general, she had a bad impression of the local government to the degree that she was reluctant to enter City Hall.

However, some institutions and people were helpful: “The school allowed us to use a healthcare room on the first floor for parent–teacher conferences.” For her child, she visited the school even though she wondered if she would be bullied by students because of her disabilities. She explained her situation to different mothers during class meetings.

#### BFP: Rejected the Value of Disability Counseling

Participant C started learning about “peer counseling” and made friends with PWD. She learned that neither PWD nor their families should be blamed for sustained disabilities, and PWD should continue presenting their opinions. She was taught how to praise her disability.

However, she disagreed with the idea that PWD were living “with pain, difficulty, and hardship, while wishing to be able-bodied. (That notion) should be denied.” She also disavowed the idea that “there were no bad PWD.” She was taught to sympathize with people without disabilities who made fun of PWD.

#### EFP: Attended a Disability Activity with People of Diverse Disabilities

Participant C joined in disability activities to learn about various issues. For example, she discovered that people with higher brain function disability are not malicious, but forgetful. She became familiar with people diagnosed with schizophrenia. She said that deaf people also have great pride. She wanted to hear from the families of PWD, having learned about the powerful solidarity among these families. “I admired their strong solidarity and that they had the power to speak passionately about their requests.” Participant C did not belong to any disability group and had to voice her views to the public.

She also wanted to learn more during disability activities about interacting with the local government because she had experienced it firsthand. She believed that a person with a disability could offer something useful to other PWD. She did not think she was suited to be a leader, speaking in front of people. She just thought “relay.” She would have liked to hand over the operations to a successor, but all other “reliable” members were busy leading activities or running other disability groups. Moreover, younger people were not interested in running activities.

#### Struggling with Managing the Activity

As a newer member, Participant C found leading the group especially challenging as the activity became interesting. Given the diversity of members’ disabilities, she had difficulties preparing for activities because she needed to find common topics in which all members would be interested. She focused on preparation for disasters such as alternative evacuation routes because the subject would affect every member; they would probably be unable to evacuate if the elevator were not working. However, she was skeptical that members would participate in these discussions.

Owing to physical limitations, Participant C was unable to perform certain tasks. She was trying to maintain her mobility because her physical strength was declining. She had trouble moving around when it was too hot, especially during summer. She could move without support less often. For these reasons, she hoped that discussions to which she contributed, summarized in the group’s monthly newsletter, would be realized as policy.

However, Participant C had a fraught relationship with local government officials because she assumed that they questioned whether PWD should focus solely on disability activities. She wanted all local officials to be involved in policy as individuals, rather than as rigid bureaucrats.

#### EFP: Attracting Successors and Increasing Member Numbers

She wanted to get others such as young people in their 30 s who are not affiliated with other disability organizations involved in disability activities. Newcomers could consult with local government agencies to find more opportunities for the younger generations.

In addition, she regarded the current leaders as reliable but reminded the members that they all had to speak out publicly about themselves. She wanted to develop members suited to leading roles. Although members were willing to be involved in activities, they were too timid to be the center of attention.

She did not think she had a solid friendship with the PWD she knew through disability activities. She said, “We are colleagues, so to speak, working together to address the same issues (such as abandoned bicycles)… but we do not have a sense of (solidarity).“ Nevertheless, she respects other members, saying “It is awesome that they are all working together to improve the (social welfare) system (considering the self-serving interests of some other PWD).”

#### P: Reacts Fearfully to the Effects of the Incident

Participant C felt that since the knife attack in Kanagawa prefecture in 2016, the general view was that PWD enjoy special treatment such as disability discounts, which include going to the movies for only 1,000 yen. She was concerned about an exaggerated discourse regarding disability assistance, in which people without disabilities were focusing on a small number who were receiving PWD benefits illegally. She also noted a trend to defend the perpetrator of the knife attack in 2016 rather than the victims, who were PWD. Moreover, Participant C had examined the public’s disapproval of PWD using buses, as she had been asked to take a bus later when it would be less crowded because she was in a wheelchair.

## Discussion

### Trajectory from Personal to Collective Aspects and Social Interests for the Next Generation

The experiences of the study’s participants were not linear recoveries; instead, they generated some ambivalence, which resulted in a series of problem-solving efforts. Their relationships with other PWD enabled them to broaden their perspectives on disability issues and realize what their activities were lacking. In the TEM, examining the unprocessed path deepens the meaning of actual experiences after the BFP (de Sousa Bastos, 2017). The BFPs involve family members, PWD, professionals, and local government officials. Considering the imagined trajectory after the BFP, the OPP, and the EFP allows for a deeper interpretation of the perception of the misalignment between people without disabilities and PWD as well as of the latter’s socially constructed deficiencies.

The onset of the disability is discussed from a perspective that differs from the biomedical model in that it considers the relationship between disease and impairment. In this study, Participant B’s relationship is unclear compared with those of Participants A and C. This perspective should be understood as the individual’s illness narrative (Kleinmann, 1988). Generally, narratives are not simple reproductions of the past, but rather reconstructions of the past that occur in the present, subject to the interference of meanings from the future. Disability studies refrain from reducing the experiences of PWD to their diseases. People with congenital disabilities have difficulty imagining themselves without their disability because it is so obvious (Bogart, 2014). Additionally, Participant B was unconcerned about her imagined life without disabilities and name of her disease or her diagnosis. Hence, her recounting of the timing of the onset of her disability reflects the abovementioned narrative traits, not her “real” experience at that time. Although Participants A and C had acquired disabilities, only Participant C clearly referred to her imagined life without disabilities. The author argues that her duration of disability was much shorter than that of Participant A.

PWD who acquire their disabilities later in life often become socially withdrawn after their discharge from hospital. While hospitalized, they are surrounded by people with similar disabilities and professionals who understand their condition. However, when they return home, they feel isolated and experience conflict with their families. *Hikikomori*, which was used by Participant A to mean social withdrawal, is a serious social problem in Japan, not limited to PWD.

However, PWD are not passive; they actively negotiate with those around them during this long and challenging period after discharge. As Valsiner et al. (2017) suggested, a person is part of an open system. For example, Participants A and C sought ways to address the burden on family members and Participant C negotiated improvements in access to meeting places with teachers. Participant B’s dilemma (the OPP and BFP) has persisted from the past to the present and into the future. Her circumstances over time have demonstrated that the reality of mild visual impairments cannot be understood through the simple distinction between visual and non-visual impairments. Additionally, Participant B highlighted the numerous challenges she has overcome in her BFPs. She navigated uncertain situations by distinguishing between those who fully understood her disability and those who did not. This distinction is a response to the school norm (SD) that requires students to pursue their studies in a manner similar to their peers. If Participant B had not experienced this dilemma, she might not have developed a sense of camaraderie with her peers at the school for the blind or become interested in the lack of agency among PWD today. In addition, if she had not attended university classes, she would not have shared her experiences as a person with a visual impairment.

Participant B mentioned that disclosing her visual impairment was to help family members and other PWD, while she rejected being seen as a special case, different from people without disabilities. Her visual impairment signaled her membership of the disability community, but it also represented an extremely personal attribute. Participant B balanced the presence of a person with a disability, which is valued in the disability community, with her personal preference for connecting with people without disabilities (Wolfensberger, 1972). In his life course study of women with congenital limb defects, Frank (1984) found the same balancing of dual attributes. Participant B’s intention was different from balancing to avoid stigma and gain support (Tagaki, 2021).

The participants recognized their physical and mental limitations due to age and hoped that younger PWD would complete their unfulfilled tasks. However, training successors involves passing on perspectives and goals, and the participants expressed frustration with PWD who do not assert themselves. Nevertheless, they tried their best. For example, Participant A was willing to let young PWD change the name of the disability center to better represent its current function. Participant B’s selective disability disclosure showed consideration for future PWD. In addition, she classified individuals by their level of activity in disability groups to identify potential leaders. Participant C believed PWD should express opinions in negotiations with local governments. She called herself a “mediator,” aspiring to engage deeply with the disability community.

If the participants had fully achieved their goals, they would have no envy or regret, and their disability experience would be complete in the EFP. However, they realized that the EFP includes paths not taken. That understanding opens the EFP to the next process through imagined paths, which include regrets, recitations, hopes, and concerns for the next generation.

### Escaping Hardship with the Value Transformation of SD and SG

All three participants sought to escape the complexity of being PWD by transforming their values in the BFPs. They re-evaluated the burden on their families, expressed sympathy for them, and shared experiences with friends who faced similar disabilities. Participant B recounted that without her kind friends, she would have gone to a school for the blind. It is argued that changing schools violated her parents’ wish for her to attend a regular school.

These transformations were influenced at multiple levels through SD and SG, which were shaped by the participants’ relationships with professionals, people without disabilities as well as PWD, social norms, and disability service and relevant policies. Sometimes, SD and SG emanate from the same source such as a professional relationship and governmental disability policy. For Participant A, living alone (the BFP) challenged the social norms of the time, which he experienced as SD. He faced the difficulty of using a wheelchair in a society unaccustomed to such individuals and had to negotiate with the local government to secure a number of care services. Those government interactions were part of his SD. Participant C also perceived negotiations with the local government as SD.

Providing SG, the public health nurse encouraged Participant A to live independently, which indirectly contributed to his support for other PWD living alone. Without their assistance, he might not have been inspired by the disability movement or formed relationships with people with diverse disabilities. Additionally, without involvement in disability-related activities, he might have felt powerless or envious of other PWD.

Participant C experienced peer counseling (the BFP) as both SD and SG. It taught her to affirm herself as a person with a disability but made her aware that the counseling perspectives might be detached from people without disabilities. If she had fully accepted the peer counseling advice, she might have unthinkingly identified with the disability community and distanced herself from people without disabilities. For her, activities with PWD and people without disabilities broadened her world and gave her satisfaction.

Similar to Participant A, Participants B and C also mentioned using disability services and remarked that several problems occurred with local governments. For example, the local government officials who did not satisfy the participants’ demands could be viewed as SD. However, encounters with these officials also motivated PWD to become interested in disability policy and the disability movement. Faced with government pushback, the three participants eagerly devoted their energies to improving the disability community for themselves and other PWD. The author argues that the local government in this context could be SG because it promotes the development of the disability community and their identity. Moreover, the Disability Discrimination Act works as SG to solve problems that impede PWD from participating in social activities. Moreover, the Act remains a resource for describing the disadvantages that PWD face in their daily lives.

SG is unexpected and random. Kind strangers helped Participant A board a train when he was struggling. In those days, PWD were not accepted in society; however, he was given support. This encounter is a typical example of a random act of kindness. However, despite the Disability Discrimination Act and media campaigns advocating for kindness toward PWD, people without disabilities are not always able to be helpful. The importance of this random act of kindness was that it provided Participant A with a resource to elaborate upon his narrative of the general attitudes toward disability.

The author argues that the participants’ narratives on SD and SG reflected the recent disability environment and general culture. The participants balanced two opposing views. The first was accepting the predominant discourse in the disability community, such as the slogan “Nothing about me without us,” and self-determination as defined in the UN Convention on the Rights of Persons with Disabilities. The second was valuing coincident SG, which reminds them that one’s achievements should not be attributed only to one’s effort, a tenet of Japanese modesty.

## Conclusion

The study’s participants undertook a series of problem-solving efforts that broadened their perspectives on disability issues, led them to realize the limitations of their own activities, and fostered their relationships with people with diverse disabilities. In the TEM, the BFP, the OPP, and the EFP are both based on social and cultural contexts, rather than a medical one. All three participants regretted not finding their successors and were not even satisfied with their achievements. However, their dissatisfaction represents an opportunity to imagine new paths and seek new activities.

The imagined trajectory after the OPP and EFP allowed them to elaborate upon the meaning of delight throughout their lives, and the BFP had personal and social significance based on the three participants’ experiences. The actual and imagined trajectories were neither better nor worse than the other. Participant C was ambivalent about her disability counseling: it empowered her but embarrassed her about her disability. The reason is that the participants actively negotiated with the people and environments surrounding them, as SD or SG; as Valsiner et al. (2017) pointed out, a person is part of an open system. SD and SG are also interchangeable depending on the perspective. SD, which hindered their activities, could be opportunities for the participants to fight against. The BFP should be interpreted with SD and SG.

Sustaining a disability identity does not require PWD to belong entirely to the disability community; instead, they alternate being inside and outside the community. For example, Participant C did not feel a sense of belonging or even any sympathy toward the members of her disability group and activities. Previous studies have shown that disability identity encompasses both personal and collective aspects as well as political and social aspects. That is valuable for people with acquired disabilities because they might suppose that being a person with a disability will lead them to fight stigma from people without disabilities (Bogart, 2014). However, the author suggests that one’s disability identity does not need to include a strong sense of belonging to the disability community if their narrative aspects of identity are used. The degree of belongingness can be flexible. Applying this dual identity concept to the participants’ narratives causes them to differ from quest narratives (Frank, 1995) and redemption narratives (McAdams & Bowman, 2001) in which PWD are expected to arrive at a goal by triumphing over adversity.

Finally, the current study implied that disability narrative research does not always need to adhere to the recognized timing of the onset of the disability (e.g., congenital vs. acquired) because, for example, Participant B was unconcerned about the timing of the onset of her disability. Additionally, Tagaki (2010) stated that people with acquired disabilities attribute their disabilities to their pre-onset lifestyle. The implication applies to narrative studies because conducting narratives involves not only remembering the past but also reconstructing experiences based on the present, as no participant’s story develops linearly from the past via the present to the future.

The limitation of the current study is that the author did not conduct interviews with any PWD born after 2000. In the past 25 years, the government has implemented many disability policies. Future studies should examine PWD’s views on such policies and the general attitude toward PWD nowadays to identify similarities and differences among generations.

## Data Availability

The data cannot be shared to protect the participants’ privacy.

## Footnotes

1 In this paper, physical disability refers to mobility; visual and hearing impairments; and internal disorders such as kidney, heart, and immunological issues based on the Act on Welfare of Physically Disabled Persons (Act No. 283 of 1949) in Japan.
